# Psychedelics assisting therapy, or therapy assisting psychedelics? The importance of psychotherapy in psychedelic-assisted therapy

**DOI:** 10.3389/fpsyg.2025.1505894

**Published:** 2025-02-05

**Authors:** Joseph A. Zamaria, Gisele Fernandes-Osterhold, Jonathan Shedler, Rachel Yehuda

**Affiliations:** ^1^Department of Psychiatry and Behavioral Sciences, University of California, San Francisco School of Medicine, San Francisco, CA, United States; ^2^Icahn School of Medicine at Mount Sinai, New York, NY, United States

**Keywords:** psychedelic-assisted therapy, psychedelics, psilocybin-assisted therapy, MDMA-assisted therapy, psychotherapy, clinical trials

## Introduction

There is notable controversy about the role of psychotherapy in clinical trials of psychedelic-assisted therapy (PAT). This heated and perpetually evolving debate informs questions such as how much therapy should accompany a psychedelic intervention (Marseille et al., 2022), what type of therapy most effectively complements psychedelic administration (Cavarra et al., 2022; Yaden et al., 2022), and even whether psychotherapy should always be included (Goodwin et al., 2023; Earleywine et al., 2024; Schenberg et al., 2024). Recent events, namely the rejection by the Food and Drug Administration (FDA) of Lykos Therapeutics' application to approve MDMA-assisted therapy (MDMA-AT) as a treatment for PTSD, has brought forth some of the intricacies within this debate. That governing body's role is to approve pharmacological agents, not psychotherapies. The FDA was likely unfamiliar with the complication of including therapy with MDMA-AT, rather than evaluating the administration of MDMA as a standalone pharmacological intervention. This highlights the novelty of PAT in medicalized settings, and underscores the controversy of including bona fide psychotherapy as a necessary component of psychedelic drug administration.

From our perspective, PAT is a unique psychiatric intervention in that it is genuinely a combined treatment, involving the administration of a psychedelic substance such as psilocybin or MDMA within the context of psychotherapy. Thus, PAT synergizes elements of pharmacological enhancement along with psychological and relational learning and healing. The application of psychedelics seems to potentiate cognitive, emotional, and behavioral change and help psychotherapy to be more effective (Cavarra et al., 2022; Grunder et al., 2024; Nayak and Johnson, 2021). Additionally, like most psychotherapies, PAT is a relational intervention. There is emerging evidence, consistent with the body of evidence in the psychotherapy literature, suggesting that the quality of the relationship between PAT therapists and patients is critically important for the success of the treatment (Levin et al., 2024; Murphy et al., 2022; Zeifman et al., 2024). The emotional impact of the real relationship developed between therapist and patient is crucial to the patient's healing, not a perfunctory safety protocol nor a peripheral afterthought.

We champion the essential role of psychotherapy within the arc of PAT in helping patients attain meaningful relief from their psychological distress, and we encourage a rigorous scientific examination of which types and doses of therapy may optimize the delivery of PAT.

## Should therapy always be a component of PAT?

While some researchers support the necessary inclusion of psychotherapy within PAT (Cavarra et al., 2022; Deckel et al., 2024; Earleywine et al., 2024; Schenberg et al., 2024), others have made a case for not including psychotherapy as a component of psychedelic administration. Goodwin et al. (2023) advance this argument, stating that there is minimal evidence to confirm the belief that PAT is a truly combined treatment and a form of enhanced or catalyzed psychotherapy. They suggest that psychedelic administration, with some psychological support for safety, is often all that is necessary to produce meaningful change for many patients suffering from serious mental health conditions, and this configuration should be the general context in which we study the effects of psychedelic administration on psychosocial health.

They first claim the type of psychotherapy used in some PAT trials has been nondirective counseling, and that nondirective counseling is not an evidence-based treatment for conditions targeted by PAT, including treatment-resistant depression, PTSD, and substance use disorders. Our perspective is that PAT is not a la carte platter, in which the “best” treatment is constructed by extracting elements of other effective non-psychedelic treatments (like evidence-based psychotherapies) and forcing them into the framework of PAT. Rather than cobbling together specific ingredients to produce an evidence-based treatment package, we advocate for the examination of which therapies are most efficacious *when delivered in concert with psychedelic administration and applied in a PAT setting*. If there is preliminary evidence that administration of psychedelics with psychological support could help treat a psychiatric condition, how might it further improve the outcome of psychedelic administration to pair it with thoughtfully-selected psychotherapy approaches, principles, or techniques that are understood to be efficacious? We will not know unless we study this phenomenon. As stated by Earleywine et al. (2024), “only data can answer this question. A clinical trial could compare groups with and without extensive preparation and integration.” The same is true regarding the position that well-trained facilitators may ensure safety and allow the patient to be fully immersed in the psychedelic experience, but that no assumptions should be made about this improving efficacy. Indeed, this assumption should not be made, but only by thoroughly studying this issue will we be able to come to a more complete understanding, preventing the urge to make assumptions.

The authors further assert that complex interactions with study facilitators, such as the kinds of interactions that exemplify most bona fide forms of psychotherapy, are an impediment to research; they complicate efforts to determine the therapeutic effect of the psychedelic, and when unregulated, they can lead to ethical violations. It becomes more difficult to account for placebo phenomenon such as expectancy, and complex patient-therapist interactions could effectively mutually unblind the treatment condition. But in PAT, the entire context of delivery—the set and setting, including the behavior of the study therapists, their interventions, and their relationships with patients—is an essential contributor to outcome. We cannot completely isolate the effects of the drug and of the psychotherapy. Separating them out might allow us to understand the impact of each aspect of PAT, but this limits our understanding of the complex interaction between these distinct avenues, when combined (Deckel et al., 2024). A similar phenomenon is true in conventional psychotherapy research; some aspects of the treatment can be randomized or dismantled, such as trials which randomize patients to two different modalities of psychotherapy, or which offer different components of a unitary treatment (e.g. randomizing patients receiving CBT to receive cognitive restructuring only or to receive behavioral activation only), but there are other aspects of therapy (like the therapist's empathy or their ability to identify and agree upon goals with patients) that are so integral to the treatment paradigm that it would be unethical or unfeasible to remove these elements in a clinical trial. Process-oriented and meta-analytic psychotherapy research has assisted in our ability to understand the therapeutic impact of these elements, even absent of a randomizable condition for them (Norcross and Lambert, 2011).

PAT should routinely include psychotherapy, and Goodwin et al.'s (2023) concerns would be addressed through methodologically rigorous research andappropriate safety protocols, along with appropriate training, regulation, and adherence monitoring of therapists to minimize the risk of ethical violations and harm to patients.

## What type of therapy should accompany PAT?

Further complicating the discussion about psychotherapy's role within PAT is the debate about the type(s) of therapy that should be employed. Current evidence is limited regarding the interaction of the modality of psychotherapy on the clinical impact of PAT, for which particular diagnoses and conditions, and for which populations of patients, but arguments have been put forth highlighting particular approaches (Brennan and Belser, 2022; Sloshower et al., 2020; Yaden et al., 2022).

### Cognitive-behavioral therapies as example

Yaden et al. (2022) argue cognitive-behavioral therapy (CBT) should be the default psychotherapy in clinical trials of PAT, citing its robust evidence base and suggesting that there is a natural match between CBT interventions and the arc of PAT. While there is an understandable temptation for researchers and clinicians to provide an orderly, structured framework for PAT, as CBT can, there are serious problems with this argument. We identify some of these problems in order to prevent a premature foreclosure of the scientific exploration of the many psychotherapy approaches which could potentially be usefully applied within PAT.

The authors suggest there is a high level of congruence between CBT concepts and the unique context of PAT. Some of these concepts, such as structured sessions and self-monitoring, may indeed be helpful for a patient receiving PAT, but there is no evidence to demonstrate that anything inherently sets them apart from any other psychotherapy techniques such that they should be the default. Other concepts referenced by the authors, such as present moment awareness, mindfulness, and reflective listening, are not unique to CBT. They have roots in humanistic-existential and psychodynamic modalities of therapy, as well as Eastern wisdom traditions, and they are employed in many varieties of psychotherapy. And yet other suggestions, such as attempting to utilize cognitive restructuring during a psychedelic administration session, may actually conflict with what is understood about the multidimensional non-ordinary states of consciousness that psychedelics can engender. Psychedelic administration sessions are often difficult to predict, and it is crucial for the facilitators to be open to the patient's process. Attempts to “control” a psychedelic experience can make the session more difficult, and could hinder the effectiveness of the session (Watts and Luoma, 2019). In psychotherapy, clinicians typically follow and support a patient's process, which is largely self-determined and sometimes open-ended. Flexibility on the part of the therapist is key. The practice of CBT, by design, does not encourage open-endedness—rather, it identifies a focus for the patient and clinician. Excessive open-ended process is typically viewed as an impediment to this focus, hijacking a specific objective. While this rationale makes sense for conventional CBT, it is not appropriate within the difficult-to-predict contours of a psychedelic session.

Most concerningly, the authors' suggestion could create a self-fulfilling prophecy. Assuming that CBT should be the *default* approach in clinical trials prematurely forecloses the scientific inquiry necessary to develop best practices and fails to acknowledge the potential effectiveness of other therapy approaches, including those commonly and historically paired with PAT, such as humanistic, depth-oriented, somatic, and transpersonal psychotherapies. Studies that could show benefits of other psychological interventions may never be conducted, or may be far lower in number than studies pairing CBT and PAT. This imbalance in research evidence accrual could sustain the false narrative that one school of psychotherapy is somehow innately superior to another, when indeed the most replicated finding in psychotherapy research is that all bona fide forms of therapy yield equivalent benefits (Budd and Hughes, 2009; Lambert, 2013; Laska et al., 2014; Shedler, 2015; Smith and Glass, 1977; Stiles et al., 1986; Wampold and Imel, 2015); CBT is “superior” only with respect to quantity of research studies, not benefits to patients compared to other bona fide psychological treatments. Elevating some PAT psychotherapies above others, without sufficient evidence, could further fracture the field, likely perpetuating infighting and misunderstanding amongst PAT therapists of differing theoretical orientations.

## Discussion

If psychotherapy is not included in PAT trials and compared with psychedelic administration with psychological support only, researchers will not be able to determine if psychotherapy is helpful, or for whom it is helpful. Additionally, we would be unnecessarily trying to disassemble a treatment known for its synergistic effect that is produced with the potentiation of psychedelic administration concurrent to the learning and processing that typically results from high-quality therapy.

In addition to PAT trials including psychotherapy, we suggest thorough research about which therapies might best synergize with PAT. This could be accomplished through head-to-head trials of PAT, with different randomized conditions corresponding with differing theoretical orientations or differing quantities of psychotherapy. Researchers also might survey patients and consumers who are potentially interested in PAT, gleaning their unique perspective and needs. This is especially important with minoritized populations who have been harmed or whose needs have not been prioritized in clinical trials of PAT (Straus et al., 2022). Finally, there would be great value in producing more studies like Levin et al. (2024), Murphy et al. (2022), and Zeifman et al. (2024), which identify the influence of common factors such as the therapeutic alliance on treatment outcome. Ultimately, the inclusion of bona fide psychotherapy in PAT, and what type, in what dose, are open questions. We value the psychological and relational impact of psychotherapy and strongly feel that this human element must be preserved and deeply examined.
